# Efficacy of intrauterine autologous blood cell derivatives in enhancing endometrial thickness and IVF outcomes for women with recurrent implantation failure: a retrospective cohort study

**DOI:** 10.1007/s10815-024-03231-5

**Published:** 2024-09-05

**Authors:** Shivangi Tiwari, Vidyashree G. Poojari, Anjali Mundkur, Prashanth Adiga, Pratap Kumar, Prashant Bhatele, Vasanthi Palanivel

**Affiliations:** 1 Department of Reproductive Medicine and Surgery, Kasturba Medical College, Manipal, Manipal Academy of Higher Education, Manipal, Karnataka India 576104; 2 Department of Neurology, Kasturba Medical College, Manipal, Manipal Academy of Higher Education, Manipal, Karnataka India 576104; 3 Seragen Biotherapeutics Pvt Ltd, Bangalore Bioinnovation Centre, Helix , Biotech Park Electronic City, Phase-1, Bangalore, India

**Keywords:** Autologous blood cell derivatives, Recurrent implantation failure, Thin endometrium, Normal endometrium, Endometrial insufficiency, Platelet-rich plasma

## Abstract

**Purpose:**

The purpose of this study was to determine the effects of intrauterine infusion of autologous blood cell derivative (ABCD) on endometrial thickness and pregnancy outcomes in a group of patients who underwent IVF with recurrent implantation failure (RIF) and who had either a normal endometrium or thin endometrium.

**Methods:**

This retrospective study included 63 patients who experienced RIF at the Department of Reproductive Medicine and Surgery, KMC, Manipal, between January 2021 and March 2024 and who received three doses of intrauterine ABCD infusion to prepare the endometrium for frozen embryo transfer (FET).

**Results:**

We enrolled 63 RIF patients, 30 with a normal endometrium (NEM) and 33 with a thin endometrium (TEM). The endometrial thickness (EMT) significantly increased across all the groups. After 3 cycles of intrauterine ABCD infusion, the mean increases in EMT in the NEM and TEM groups were 0.77 mm and 1.36 mm, respectively, which were statistically significant. Among the 62 completed FET cycles, 40.3% were positive for beta-hCG. The clinical pregnancy rate was 33.8% (40% in the NEM group, 28.1% in the TEM group), and the live birth rate was 24.2% (30% in the NEM group, 18.8% in the TEM group). A total of 9.7% of pregnancies had spontaneous miscarriages. Moreover, the EMT did not differ between the pregnant and nonpregnant groups.

**Conclusion:**

Intrauterine ABCD infusion improves the pregnancy outcomes of patients with RIF, regardless of the EMT. The results of this study revealed that endometrial receptivity improved significantly along with the EMT.

**Supplementary Information:**

The online version contains supplementary material available at 10.1007/s10815-024-03231-5.

## Introduction

Recurrent implantation failure RIF is a disorder characterized by the failure of high-quality embryos to attach successfully to the endometrium after multiple attempts at IVF cycles [1]. RIF affects approximately 10% of patients with IVF worldwide [2, 3]. There is a disagreement among experts over the exact definition of the RIF. The definition of RIF relies mostly on the number of failed treatment cycles and embryos transplanted. RIF is defined as two consecutive failed cycles of embryo transfer involving good-quality embryos. This definition helps in detecting possible problems with implantation at an earlier stage and enables prompt intervention [4]. However, some experts recommend a higher threshold for RIF, defined as three or more failed cycles, to limit the possibility of false-positive diagnoses and unnecessary treatment [5, 6]. A recent survey revealed that most clinicians consider three or more unsuccessful ETs of good-quality embryos as RIF, matching the European Society of Human Reproduction and Embryology (ESHRE)-preimplantation genetic diagnosis (PGD) consortium definition, while also considering the patient’s age [7].

RIF has multiple causes, including immunological variables, uterine factors such as chronic endometritis, a thin endometrium, poor endometrial quality, an incompatible implantation window and embryonic factors such as decreased blastocyst competency and a lack of embryo–endometrial synchronization [8]. Although both uterine and embryonic variables are commonly acknowledged as important factors in RIF, endometrial receptivity plays a vital role. Endometrial receptivity is critical for effective embryo implantation, as evidenced by the low implantation rate of euploid blastocysts (< 70%) [9]. Approximately two-thirds of implantation failures are attributed to subpar endometrial receptivity and insufficient embryo–endometrial communication [10].

TEM is the most significant contributor to implantation failure and is associated with an elevated risk of abortion [11]. TEM is associated with reduced pregnancy rates, spontaneous abortion, ectopic pregnancies, improper placentation, and obstetric challenges [12–15]. Studies have shown that an EMT of less than 7 mm occurs in 0.7 to 2.5% of fresh IVF cycles. The exact incidence of frozen IVF cycles has not been widely reported because they are frequently cancelled [16–20]. This can be explained by inadequate epithelial growth, poor vascular development, and increased blood flow resistance in the uterine radial arteries, which leads to decreased expression of the vascular endothelial growth factor (VEGF) receptor [21, 22].

Hence, the condition of the endometrium is crucial for successful implantation of an embryo. A variety of circumstances, such as injury, loss of receptivity, or lack of proliferation, can cause endometrial insufficiency. Studies have shown a clear correlation between EMT and the likelihood of successful embryo implantation. Therefore, it is normal in clinical practice to refrain from performing embryo transfer if the patient’s EMT is less than 7 mm [20, 23].

Despite the reported efficacy of prolonged estrogen therapy [24], aspirin [25], vitamin E, transvaginal endometrial perfusion of granulocyte colony-stimulating factor (G-CSF) [26], vaginal sildenafil citrate application [27], vitamin C and E, and L-arginine supplementation [28, 29], steroid therapy [30], and endometrial scratching [31, 32] in improving the EMT, a significant number of women with TEM do not respond to these treatments. To resolve problems related to the compatibility and asynchronicity between the endometrium and embryo, attempts have been made to analyze endometrial receptivity throughout different phases of the reproductive cycle. Although some achievements have been made in identifying abnormal receptivity status, new research has raised doubts about the effectiveness of receptivity profiling [33]. Some researchers have suggested that these techniques are intrusive and have limited practicality. Furthermore, there is a paucity of effective treatments for those with poor receptivity and TEM results [34, 35]. Genetic and recombinant engineering techniques can generate growth factors, although this approach appears to be more costly and time-consuming. Furthermore, multiple doses are needed to achieve optimal therapeutic outcomes.

Platelets are rich in growth factors that promote proliferation and development, including platelet-derived growth factor (PDGF), VEGF, epidermal growth factor, and transforming growth factor. Studies have indicated that, in reproducing the natural processes of tissue repair and regeneration, PDGF is both safe and efficient [36]. PDGFs are essential for a variety of processes, such as angiogenesis, cell proliferation, and tissue repair. They increase VEGF expression, stimulate the growth and movement of endothelial cells and vascular smooth muscle cells, and regulate the synthesis and degradation of matrix components to regulate the extracellular matrix [37]. PDGFs are present in the endometrium, with the most elevated levels observed during the proliferative phase of the menstrual cycle, indicating endometrial regeneration [38].

An innovative and hopeful treatment involves the administration of ABCD through intrauterine infusion. ABCD preparation, which involves centrifuging the patient’s blood to increase the platelet concentration, is hypothesized to stimulate endometrial proliferation, induce neoangiogenesis, and have anti-inflammatory effects to improve implantation success [39, 40].

The autologous approach is devoid of any detrimental side effects and is non-allergenic. It diminishes the likelihood of immunological reactions and the spread of infections, offering a preferable substitute for medical treatment. This therapy involves the extraction of blood from the patient during a period of rapid cell division and subsequent treatment to increase the concentration of platelets in the plasma. The procedure involves the collection of red and white blood cells, yielding a small amount (0.3–1.0 cc) of ABCD. One to 5 days before embryo transfer, the uterine cavity receives an injection of this ABCD agent. Endometrial growth has been the most commonly reported impact of ABCD intrauterine treatment in patients with thin endometria during ART cycles. Angiogenesis is an important process that helps the endometrium grow after a period of time. For implantation to occur, the endometrium needs to be well vascularized and open to the embryo. Hence, the presence of growth factors and other cytokines in ABCD fluid may stimulate the thickening of the endometrium in individuals with a thin endometrium [41, 42].

A preliminary study revealed substantial improvements in the EMT, embryo implantation, and clinical pregnancy for patients with both a TEM and a NEM who continue to fail to become pregnant [43].

However, reports on the effect of ABCD infusion on women without a TEM are currently limited and restricted to a few centers. Thus, we aimed to retrospectively investigate the effects of intrauterine ABCD infusion on EMT and IVF outcomes in patients with RIF and compare the outcomes between patients with RIF with a NEM and those with a TEM.

## Materials and methods

### Study population

This retrospective cohort study included infertile patients with RIF with either a NEM or TEM who received ABCD treatment to prepare the endometrium for FET at the Department of Reproductive Medicine and Surgery, Kasturba Medical College, Manipal, a referral center in southern India, between January 2021 and March 2024. This study included patients with RIF (characterized as having a history of implantation failure during at least two consecutive FET cycles using good-quality embryos), who had three cycles of intrauterine ABCD infusions and normal transvaginal ultrasonography results. RIF patients were classified into two categories. The NEM group comprised patients with a normal endometrium; the TEM group comprised those with a thin endometrium. Patients were considered to have a TEM if their endometrium was consistently thin (< 8 mm) during at least two previous embryo transfer cycles, even if good-quality embryos were transferred [4, 44]. We excluded patients with hemoglobin levels less than 11 g/dl, platelet counts less than 15 million/mm^3^, hepatic disorders, HIV, active lower genital tract infections, genetic abnormalities, hematologic disorders, autoimmune disease, or congenital uterine anomalies.

All patients were provided with comprehensive information regarding ABCD treatment before their FET cycle commenced and made their own decisions regarding whether to undergo ABCD therapy. Written informed consent for blood sampling and intrauterine ABCD infusion was obtained from all the participants. Before the procedure, all the participants underwent blood tests for antiphospholipid antibodies (APLAs). Patients with persistent TEM in previous transfer cycles had undergone hysteroscopic examination to confirm the absence of anatomical abnormalities and to rule out tuberculosis, which is prevalent in India. Ethical approval was granted by the Kasturba Medical College and Kasturba Hospital Institutional Ethics Committee (IEC1–224–2024, approval date 2 July 2024), and we conducted this study in accordance with the ethical principles of the Declaration of Helsinki.

### ABCD preparation

We produced three doses of ABCD growth factor concentrate from 30 mL of peripheral blood. The ABCD growth factor concentrate was created by concentrating platelets from fresh peripheral blood obtained from a vein during the proliferative phase, storing them in anticoagulant, and processing them to separate distinct blood components. Autologous blood was processed to isolate a concentrated platelet fraction while keeping lymphocytes and red blood cells (RBCs) less than 1%. The preparation technique was divided into two centrifugation phases, with optimization of the temperature, centrifugal force, and time. The first centrifugation step separates RBCs and the buffy coat from platelet-rich plasma (PRP). The second centrifugation procedure, however, focuses on capturing platelets in the lower phase, whereas the top phase contains platelet-poor plasma (PPP). The platelet count in whole blood and the platelet-rich fraction (PRF) were assessed via an automatic blood tester and immunophenotyping with antibodies specific to CD61, CD63, P selectin, and an early-stage platelet activation marker (Beckman Coulter PK7400 Automated Microplate System Analyzer). A proprietary filter was used to extract ABCD from platelet concentrate induced to produce growth factors and anti-inflammatory cytokines via the selective growth factor enrichment protocol of Seragen.

We transferred the upper fraction of whole blood into a sterile tube without disrupting the RBC layer, following a proprietary protocol for selective enrichment on the basis of centrifugation. We partitioned a sample volume of 100 μl to determine the concentration and purity of the platelets. After collection, the upper fraction was centrifuged for 12 min. We sterilely transferred PPP into a tube. The obtained platelet particles were reconstituted in PPP. As a result of the aforementioned separations, the platelet fraction was concentrated even more, and platelets were induced to secrete cytokines and growth factors. After centrifugation at 3000 × g for 20 min at 18 °C for recovery, we aliquoted the enriched growth factor concentrate (ABCD) into three 1 ml volumes with PPP. Approximately 600 × 106 platelets were utilized in the preparation of the growth factor concentrate at their final concentration [40, 45].

### Endometrial preparation and embryo transfer protocol

We treated patients with autologous ABCD agents in estrogen-primed FET cycles. The endometrial preparation involved the use of oral estradiol valerate at a dosage of 4 mg/day starting on days 2–3 of the menstrual cycle, which was increased to a maximum dosage of 12 mg/day in cases of inadequate endometrial growth (< 7 mm). ABCD was administered via an intrauterine insemination catheter. We administered the first dose of ABCD on days 5, 6, or 7 of the menstrual cycle or when the bleeding stopped completely after providing informed consent, and doses two and three were frozen for future use. We administered the second dose 5 days after the first dose and the third dose 2 days before embryo transfer. Bed rest was not required after the procedure. The patient who was positive for APLA received low-molecular-weight heparin. EMT was measured by transvaginal ultrasonography on the 1st, 2nd, and 3rd doses of ABCD infusion. The administration of transvaginal progesterone supplementation (8% twice daily) was performed when the endometrium exhibited a trilaminar appearance and a thickness above 7 mm.

Embryo transfer to the uterine cavity was performed either 3 days (day 3 embryo) or 5 days (day 5 embryo) after the initial progesterone administration. Assessment of embryo quality was performed both prior to freezing and post thawing before transfer. The quality of the embryos was assessed via the Istanbul Consensus grading system. Grade 1 and 2 cleavage-stage embryos denote high-quality cleavage and are considered suitable for use. Grade 1 blastocysts were classified as high quality, whereas grade 1 and grade 2 blastocysts were deemed suitable for transfer [46].

**Fig. 1:**
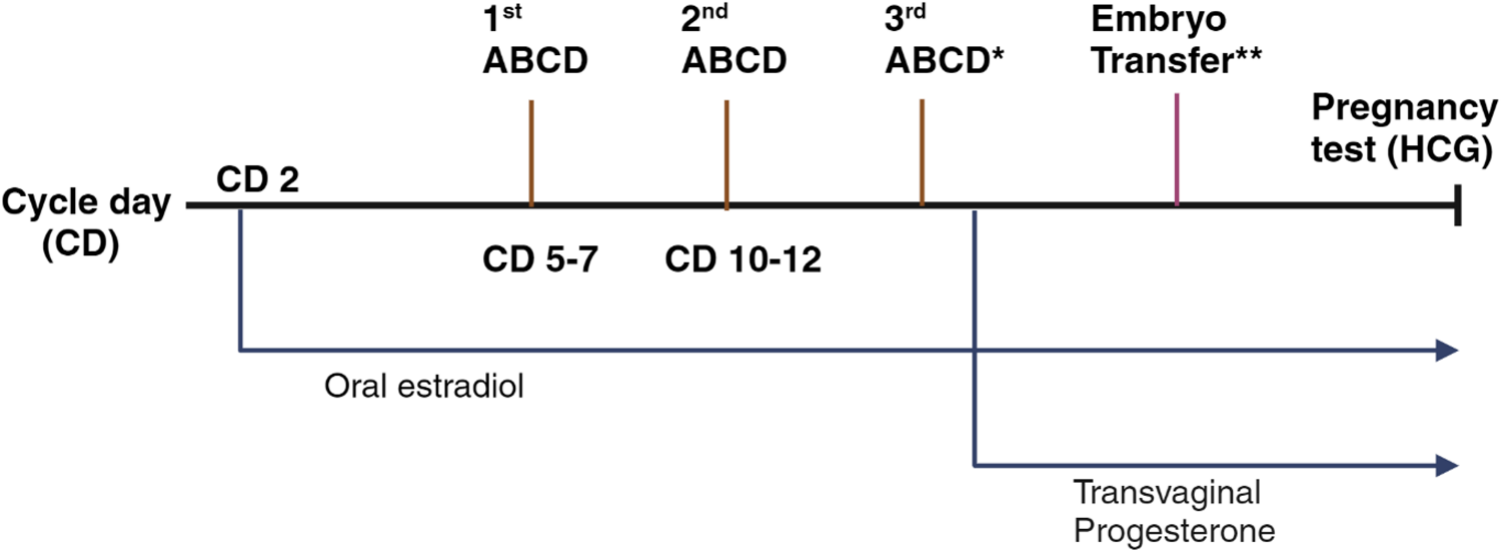
The chronological sequence of the embryo transfer cycle protocol, including intrauterine ABCD infusion. The first dose of ABCD was given on days 5, 6, or 7 of the menstrual cycle or after the bleeding had completely stopped, and the second dose was given 5 days later. *A third dose of ABCD infusion was given 2 days before embryo transfer. The endometrial preparation involved the use of oral estradiol valerate at a dosage of 4 mg/day beginning on days 2–3 of the menstrual cycle. When the endometrium appeared trilaminar and had a thickness of more than 7 mm, transvaginal progesterone supplementation (8% twice daily) was administered. **Embryonic transfer to the uterine cavity occurred either 3 days (day 3 embryo) or 5 days (day 5 embryo) after the initial progesterone treatment. The serum β-hCG concentration was measured 14 days after embryo transfer. Abbreviations: ABCD, autologous blood cell derivative; CD, cycle day; HCG, human chorionic gonadotropin

Biochemical pregnancy was determined by the serum β-hCG concentration at 14 days after embryo transfer. After 14 days of embryo transfer, we considered the serum β-hCG concentration to be positive if it exceeded 5 mIU/mL. Clinical pregnancy was determined by the presence of a gestational sac through transvaginal ultrasonography. Estradiol supplementation was continued until the seventh week of gestation, and progesterone supplementation was continued until the twelfth week of gestation. Figure 1 depicts the chronological sequence of the embryo transfer cycle protocol, including intrauterine ABCD infusion.

### Statistical analysis

The data were entered into a Microsoft Excel spreadsheet. Categorical variables are expressed as numbers and percentages, whereas continuous variables are expressed as the mean ± standard deviation (SD) and variance. The text presents differences in EMT as the mean, standard deviation, variance, and median plus (interquartile range). Pearson’s chi-square test was used to compare categorical datasets between the NEM and TEM groups, whereas Student’s *t* test was used for continuous datasets. The Kolmogorov‒Smirnov test for normality was used to assess the normal distribution of the EMT. A paired *t* test was used for assessing the means pre- and postintervention, whereas an unpaired *t* test was used to compare the means of two independent groups. Single and multivariate logistic regression analyses were performed to identify predictive factors influencing the therapeutic outcomes of ABCD-FET, including β-hCG positivity, clinical pregnancy, and live birth. All the tests were two-tailed, and *P* < 0.05 indicated statistical significance. SPSS version 25.0 was used for the statistical analysis.

## Results

### Patient demographics

**Fig. 2:**
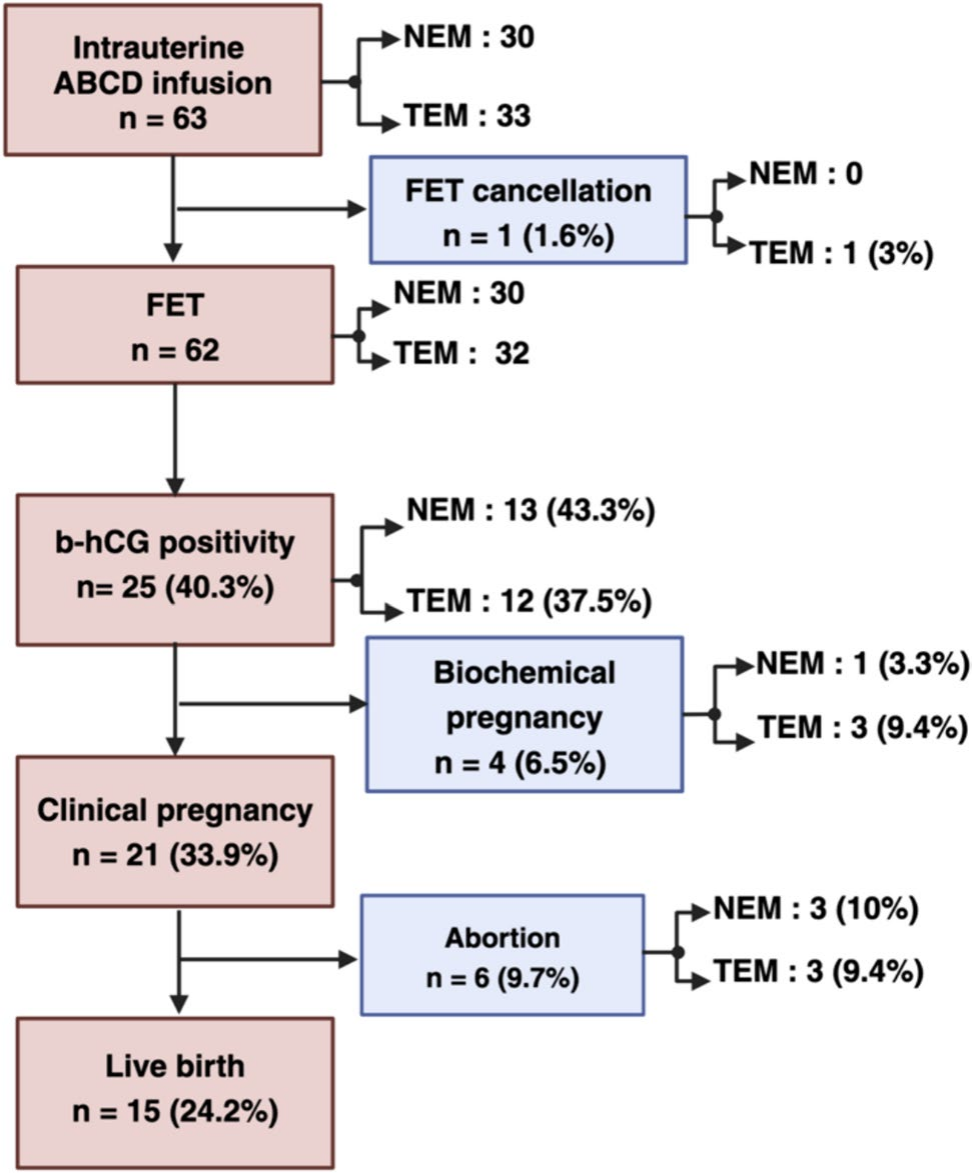
Study flow chart showing the participation of patients throughout the study. The numbers of participants are shown. Abbreviations: ABCD, autologous blood cell derivative; FET, frozen embryo transfer; HCG, human chorionic gonadotropin; NEM, normal endometrium; TEM; Thin endometrium

After providing informed consent, the analysis included 63 patients who received three doses of ABCD in accordance with the protocol (Fig. 2). None of the patients met the exclusion criteria. The age range of the patients was 25–45 years, with an average of 34.90 ± 5.75 years and an average BMI of 24.27 ± 4.42 kg/m^2^. Among the patients, 44.4% (*n* = 28) had primary infertility, and 55.6% (*n* = 35) had secondary infertility. The mean duration of infertility ranged from 2 to 26 years, and the average duration was 7.71 ± 4.36 years. The most common reason for infertility was a decrease in the ovarian reserve (DOR) (27% of patients). The type of IVF was self-IVF in 65.1% of the patients (*n* = 41). The highest number of previous IVF failures reported was one, which occurred in 26 (46.3%) patients. The mean number of previous implantation failures was 2.59. Two embryos were transferred per patient, accounting for 44 (71%) embryos. APLAs tested positive in one patient (3.3%) in the NEM group and eight patients (24.2%) in the TEM group, which was a significant difference. Except for the prevalence of APLA positivity, there was no significant difference in age, BMI, duration of infertility, reason for infertility, type of infertility, type of IVF, past IVF failure, or previous implantation failure between these two groups (Table 1).

**Table 1:** Characteristics of the participants in the NEM and TEM groups

Baseline characteristics	NEM group (*n* = 30) Mean ± SD, variance	TEM group (*n* = 33) Mean ± SD, variance	Total (*n* = 63) Mean ± SD, variance	*P* value
Age (year)	33.93 ± 5.55, 30.82	35.79 ± 5.58, 15.59	34.90 ± 5.75, 33.15	0.20
BMI (kg/m^2)^	23.68 ± 3.95, 15.59	24.80 ± 4.81, 23.11	24.27 ± 4.42, 19.54	0.32
Infertility duration (year)	7.43 ± 3.80, 14.46	7.97 ± 4.86, 23.65	7.71 ± 4.36, 19.04	0.63
Previous implantation failure	2.57 ± 0.73, 0.53	2.61 ± 0.97, 0.93	2.59 ± 0.85, 0.73	0.85
	*n*, %	*n*, %	*n*, %	
Type of infertility				
Primary Secondary	16, 53.3 14, 46.7	12, 36.4 21, 61.8	28, 44.4 35, 55.6	0.17
Causes of infertility				
1: Endometriosis 2: Ovulation 3: Unexplained 4: Male 5: DOR 6: Tubal 7: Combined	1, 3.3 6, 20 6, 20 5, 16.7 6, 20 4, 13.3 2, 6.7	4, 12.1 7, 20.6 4, 12.1 3, 9.1 11, 33.3 3, 9.1 1, 3.0	5, 7.9 13, 20.3 10, 15.9 8, 12.7 17, 27.0 7, 11.1 3, 4.8	0.59
APLA positivity	1, 3.3	8, 24.2	9, 14.3	0.01
Types of IVF				
Self Donor	22, 73.3 8, 26.7	19, 57.6 14, 42.4	41, 65.1 22, 34.9	0.19
Previous IVF failures				
0 1 2 3 4	11, 36.7 13, 43.3 5, 16.7 1, 3.3 0, 0	14, 42.4 13, 39.4 4, 12.1 1, 3.0 1, 3.0	25, 39.7 26, 41.3 9, 14.3 2, 3.2 1, 1.6	0. 85
Previous implantation failures				
2 3 4 5 6	16, 53.3 12, 40.0 1, 3.3 1, 3.3 0, 0	20, 60.6 9, 27.3 2, 6.1 1, 3.0 1, 3.0	36, 57.1 21, 33.3 3, 4.8 2, 3.2 1, 1.6	0.72

Categorical datasets were compared via Pearson’s chi-square test, and continuous datasets were compared between the NEM and TEM groups via Student’s *t* test. *APLA* antiphospholipid antibodies, *BMI* body mass index, *CI* confidence interval, *DOR* decreased ovarian reserve, *IVF* in vitro fertilization, *NEM* normal endometrium, *RIF* recurrent implantation failure, *SD* standard deviation, *TEM* thin endometrium

### Endometrial proliferation in response to ABCD

The mean EMT on the day of the first intrauterine infusion of ABCD agent was 9.08 ± 0.76 mm in the NEM group and was significantly greater than that in the TEM group (6.96 ± 0.76 mm). After three cycles of intrauterine infusion of ABCD, there was a significant increase in the EMT, with an average of 9.85 ± 1.41 in the NEM group and 8.35 ± 1.19 in the TEM group. Before and after ABCD infusion, the mean differences in EMT between the NEM and TEM groups were 2.12 mm and 1.53 mm, respectively, which were significantly decreased. The EMT significantly increased across all the groups. The NEM and TEM groups presented statistically significant increases in the mean EMT of 0.77 mm and 1.36 mm, respectively, after three cycles of ABCD infusion (Table 2). EMT followed a normal distribution in both the NEM and TEM groups. (Supplementary Table 1) Following the administration of ABCD, there was no significant difference in EMT between the NEM and TEM groups, as determined by the clinical pregnancy status. The increase in EMT before and after ABCD infusion did not differ between the pregnant and nonpregnant groups (*p* values of 0.51 and 0.12, respectively) (Table 3).

**Table 2:** Changes in endometrial thickness before and after an infusion of ABCD in the NEM and TEM groups

Endometrial thickness (mm)	Total (*n* = 63) Mean ± SD	NEM group (*n* = 30) Mean ± SD, 95% CI	TEM group (*n* = 33) Mean ± SD, 95% CI	Mean difference (95% C.I)	*P* value
The day of the first ABCD infusion	7.97 ± 1.31	9.08 ± 0.76; 8.80, 9.37	6.96 ± 0.76; 6.69, 7.23	2.12 (1.73,2.50)	< 0.01
The day of the second ABCD infusion	8.80 ± 1.59	9.79 ± 1.33; 9.29, 10.29	7.90 ± 1.24; 7.46, 8.35	1.88 (1.23,2.54)	< 0.01
The day of the third ABCD infusion	9.06 ± 1.45	9.85 ± 1.41; 9.29,10.37	8.35 ± 1.19; 7.99, 8.73	1.53 (0.84, 2.10)	< 0.01
Mean difference (95% C.I) *P* value		0.77 (0.22, 1.28) 0.005	1.36 (1.01, 1.70) < 0.001		

The mean endometrial thickness between the NEM and TEM groups was compared via Student’s *t* test. *ABCD* autologous blood cell derivative, *CI* confidence interval, *NEM* normal endometrium, *TEM* thin endometrium

**Table 3:** EM and TEM groups, based on clinical pregnancy outcomes

Endometrial thickness (mm)	NEM group Success (*n* = 12), failure (*n* = 18), *P* value	TEM group Success (*n* = 9), failure (*n* = 23), *P* value	
The day of the first ABCD infusion	9.04 ± 0.95, 9.12 ± 0.64, 0.79	7.18 ± 0.73, 6.93 ± 0.75, 0.39	
The day of the second ABCD infusion	9.52 ± 1.11, 9.98 ± 1.47, 0.37	8.01 ± 0.49, 8.05 ± 1.14, 0.92	
The day of the third ABCD infusion	9.66 ± 0.96, 9.95 ± 1.72, 0.61	8.23 ± 0.67, 8.50 ± 1.05, 0.57	
Endometrial thickness (mm)	Pregnant group (*n* = 21) Mean ± SD, variance	Non pregnant group (*n* = 41) Mean ± SD, variance	*P* value
Pre-ABCD infusion	7.88 ± 1.26, 1.61	7.88 ± 1.30, 1.69	0.51
Post-ABCD infusion	9.08 ± 1.08, 1.18	9.14 ± 1.55, 2.39	0.12

The mean endometrial thickness of patients with successful and failed clinical pregnancy cycles was compared via Student’s *t* test. The mean endometrial thickness between the pregnant and nonpregnant groups was compared via Student’s *t* test. *ABCD* autologous blood cell derivative, *NEM* normal endometrium, *SD* standard deviation, *TEM* thin endometrium

### Embryo transfer outcomes

The number of embryos transferred did not differ significantly between the groups. In the TEM group, one FET cycle was terminated because of abnormal uterine bleeding. Among the 62 FET cycles that progressed, the majority of embryos transferred were two-thirds (73.3%) in the NEM group and 68.8% in the TEM group. After three cycles of ABCD infusion, the beta HCG positivity rate (hCGR) was 43.3% (13/30) in the NEM group and 37.5% (12/32) in the TEM group; the biochemical pregnancy rate was 3.3% (1/30) in the NEM group and 9.4% (3/32) in the TEM group; the clinical pregnancy rate (CPR) was 40% (12/30) in the NEM group and 28.1% (9/32) in the TEM group; and the live birth rate (LBR) was 30% (9/30) in the NEM group and 18.8% (6/32) in the TEM group. There were no reports of ectopic pregnancies among patients in either group. Among the 21 patients who achieved clinical pregnancies, three patients in each group experienced abortion. The hCGR, CPR, and LBR were greater in the NEM group than in the TEM group. Nevertheless, there were no significant differences in the hCGR, CPR, or LBR between the NEM and TEM groups (Table 4). Furthermore, there was no difference in the Istanbul grade of transferred embryos between participants who achieved clinical pregnancy and those whose pregnancies failed in the TEM or RIF groups throughout the treatment cycle (Table 5).

**Table 4:** Embryo outcomes in the NEM and TEM groups

Characteristics	NEM group (*n* = 30) *n*, %	TEM group (*n* = 32) *n*, %	Total (*n* = 62) *n*, %	*P* value
Embryo transferred				
1 2 3	7, 23.3 22, 73.3 1, 3.3	9, 28.1 22, 68.8 1, 3.1	16, 25.8 44, 71.0 2, 3.2	0.91
FET cancellation		1, 3.0	1, 1.6	0.34
Beta HCG positivity	13, 43.3	12, 37.5	25, 40.3	0.64
Biochemical pregnancy				
Positive Negative	1, 3.3 29, 96.7	3, 9.4 29, 90.6	4, 6.5 58, 93.5	0.33
Clinical pregnancy				
Yes No	12, 40 18, 60	9, 28.1 23, 71.9	21, 33.9 41, 66.1	0.32
Abortion				
Yes No	3, 10 27, 90	3, 9.4 29, 90.6	6, 9.7 56, 90.3	0.93
Live births				
Yes No	9, 30 21, 70	6, 18.8 26, 81.3	15, 24.2 47, 75.8	0.30

Categorical datasets were compared via Pearson’s chi-square test. *ABCD* autologous blood cell derivative, *FET* frozen embryo transfer, *HCG* human chorionic gonadotropin, *NEM* normal endometrium, *SD* standard deviation, *TEM* thin endometrium

**Table 5:** Morphology of the transferred embryos in the NEM and TEM groups, according to their clinical pregnancy status

Embryo grading	NEM group Success (*n* = 12), failure (*n* = 18) *n*, %, *n*, %	*P* value	TEM group Success (*n* = 9), failure(*n* = 23) *n*, %, *n*, %	*P* value
Day 3				
Grade 1 Grade 2	7, 58.3, 9, 50.0 0, 0, 5, 27.8	0.20	6, 66.7, 11, 47.8 0, 0, 6, 26.1	0.31
Day 5				
Grade 1 Grade 2	3, 25.0, 3, 16.7 2, 16.7, 1, 5.6		3, 33.3, 5, 21.7 0, 0, 1, 4.3	

Note: Categorical datasets were compared via Pearson’s chi-square test. Abbreviations: *NEM* normal endometrium, *TEM* thin endometrium

We performed univariate and multivariate logistic regression analyses to identify factors contributing to beta-hCG positivity, clinical pregnancy, and live births. The explanatory variables included age, BMI, number of previous implantation failures, final EMT, and embryo quality. No significant associations were detected between the hCGR, CPR rate, or LBR in either group (Table 6).

**Table 6:** Factors associated with the success of ABCD-FET in the NEM and TEM groups

Characteristics	NEM group				TEM group			
	Crude OR	*P* value	Adjusted OR (95% C.I)	*P* value	Crude OR	*P* value	Adjusted OR (95% C.I)	*P* value
Beta HCG positivity								
Age (years)	0.91	0.20	0.91 (0.78, 1.07)	0.28	0.99	0.93	0.99 (0.87, 1.12)	0.93
BMI (kg/m^2^)	0.99	0.77	0.94 (0.76, 1.16)	0.57	0.98	0.79	0.98 (0.83, 1.15)	0.79
No. of previous implantation failures	0.34	0.13	0.34 (0.08, 1.37)	0.13	0.86	0.71	0.86 (0.39, 1.90)	0.71
Final EMT (mm)	0.87	0.66	0.87 (0.47, 1.60)	0.66	0.80	0.58	0.80 (0.36, 1.76)	0.58
Embryo grading	1.96	0.11	0.51 (0.23, 1.16)	0.10	0.52	0.15	1.95 (0.78, 4.89)	0.15
Clinical pregnancy								
Age (years)	0.96	0.64	0.96 (0.82, 1.12)	0.64	1.1	0.26	1.08 (0.94, 1.24)	0.26
BMI (kg/m^2^)	0.84	0.15	0.84 (0.67, 1.07)	0.15	1.0	0.96	1.0 (0.84, 1.19)	0.96
No. of previous implantation failures	0.35	0.15	0.35 (0.08, 1.44)	0.15	0.89	0.79	0.89 (0.38, 2.09)	0.79
Final EMT (mm)	0.93	0.81	0.92 (0.50, 1.69)	0.81	0.79	0.58	0.79 (0.34, 1.83)	0.58
Embryo grading	1.50	0.30	1.50 (0.68, 3.27)	0.30	0.74	0.52	0.74 (0.30, 1.83)	0.52
Live births								
Age (years)	1.04	0.62	1.04 (0.89, 1.21)	0.62	1.02	0.76	1.02 (0.87, 1.19)	0.76
BMI (kg/m^2^)	0.91	0.43	0.91 (0.72, 1.15)	0.43	1.01	0.92	1.01 (0.83, 1.23)	0.92
No. of previous implantation failures	0.44	0.27	0.44 (0.10, 1.93)	0.27	0.31	0.27	0.31 (0.04, 2.49)	0.27
Final EMT (mm)	0.82	0.57	0.82 (0.43, 1.59)	0.57	0.90	0.85	0.90 (0.30, 2.69)	0.85
Embryo grading	2.23	0.07	2.23 (0.93, 5.35)	0.07	1.42	0.50	1.42 (0.50, 4.03)	0.51

The crude ORs were generated via binary logistic regression analyses, and the adjusted ORs were generated via multivariate logistic regression analysis. *ABCD* autologous blood cell derivative, *BMI* body mass index, *CI* confidence interval, *EMT* endometrial thickness, *HCG* human chorionic gonadotropin, *NEM* normal endometrium, *OR* odds ratio, *RIF* recurrent implantation failure, *TEM* thin endometrium

## Discussion

The introduction of IVF has greatly influenced the increase in pregnancy rates, leading to notable advancements in reproductive medicine. At present, there is no universally agreed upon treatment for patients with RIF who have a normal or thin endometrium. ABCD has now been introduced into the field of reproductive medicine. ABCD developed from autologous blood provides a direct and noninvasive method for delivering concentrated growth factors and cytokines to the endometrium. ABCD research has yielded positive results in women with thin endometria, DOR, or RIF.

The main objective of this study was to determine the effects of ABCD growth factor concentrate on IVF outcomes in patients with RIF with a NEM and a TEM. In this study, we administered ABCD growth factor concentrate to 63 patients. Sixty-two patients underwent FET. One patient in the TEM group experienced FET cancellation due to abnormal uterine bleeding.

Endometrial receptivity is crucial for embryo implantation. The EMT serves as a reliable measure of endometrial receptivity and provides valuable information about the likelihood of a successful pregnancy after embryo transfer. There is ongoing controversy about the precise definitions of the TEM. Most studies consider a TEM to be either < 7 mm or < 8 mm. One study indicated that patients with an EMT < 8 mm after their first FET cycle had decreased CPR and LBR. Further review of the Canadian IVF database revealed a reduced live birth risk for those with an EMT < 7 mm [44, 47]. Nevertheless, two studies conducted on patients who received oocyte donation together with hormone replacement cycles revealed that the thickness of the endometrium does not impact the rate of conception [48, 49].

We defined a TEM with a thickness of less than 8 mm in this study. In this retrospective study of RIF patients, we observed significant increases in the EMT after three cycles of ABCD infusions in all our patients. When comparing pre-ABCD infusion EMT within and between the NEM and TEM groups, post-ABCD infusion EMT was significantly greater even after a single cycle of ABCD infusion, suggesting that the endometrial proliferation observed in both groups was mostly due to ABCD therapy.

Chang et al. first demonstrated the effectiveness of intrauterine PRP injection in treating RIF and thin endometrial infertility [50]. Tandulwadkar et al. investigated 68 women who underwent TEM with more than two cycles of cancellations and reported that the mean pre-PRP EMT was 5 mm, which rose significantly to 7.22 mm after PRP treatment [51]. In a study of 19 patients with refractory TEM, Molina et al. reported that a second PRP infusion led to an increase in the EMT of more than 9 mm [52]. Eftekhar et al. conducted the first randomized controlled trial (RCT) involving 66 subjects with TEM. PRP treatment led to a substantial increase in EMT compared with that in the control group, indicating its potential to improve endometrial responses [53]. Nazari et al. reported similar results in a double-blind, randomized, sham-controlled trial of 60 women whose FET cycles were cancelled due to insufficient EMT [54]. Kusumi et al. reported that administering PRP increased EMT by 1.27 mm in women with RIF compared with the previous embryo transfer cycle [55]. According to a retrospective analysis by Coksuer et al., EMT increased significantly at 48 h after PRP therapy compared with before PRP therapy (10 mm versus 6.25 mm) [56]. The mean differences in EMT between the 4 RCTs with 307 participants and the 9 non-RCTs with 675 participants were 0.93 and 1.16, with 0.59–1.27 and 0.68–1.65 95% CIs, respectively [57]. Fuji et al. conducted a study that included 41 RIF patients and 64 TEM patients. The second PRP infusion cycle resulted in substantial increases in EMT, with the former achieving an average thickness of 9.4 ± 0.2 mm and the latter reaching an average thickness of 7.4 ± 0.2 mm [58].

Our findings are in line with those of previous studies showing the effectiveness of intrauterine PRP infusion in RIF patients. The intervention resulted in a statistically significant improvement in EMT, with averages of 9.85 ± 1.41 in the NEM group and 8.35 ± 1.19 in the TEM group. The mean increases in EMT across the NEM and TEM following three cycles of ABCD infusion were 0.77 mm and 1.36 mm, respectively, and were statistically significant.

Russel et al. reported that intrauterine PRP infusion one or more times throughout a cycle increased the median EMT from 6.7 (IQR 1.0) to 7.6 mm (IQR 1.0), regardless of the diagnosis of RIF or TEM. The median EMT increased from 6.5 (IQR 1.0) to 7.3 mm (IQR 1.3) in all groups after a single PRP infusion, with statistically significant increases in each group [59]. The current findings also exhibited a similar trend. After three cycles of intrauterine ABCD infusion, the median EMT increased from 7.9 (IQR 1.9) to 9.0 mm (IQR 1.8), regardless of EMT. Furthermore, following a single PRP infusion, all groups showed a statistically significant increase in the median EMT from 7.9 (IQR 1.9) to 8.6 mm (IQR 2.1).

It has been hypothesized that the primary effect of ABCD on the endometrium may involve functional rather than structural properties. Kuroda et al. reported that intrauterine PRP infusion promotes tissue healing through a transitory inflammatory response, cell growth, and antimicrobial action during endometrial decidualization, but these effects are reduced in undifferentiated human embryonic stem cells [60]. ABCD regulates the processes of cell growth, tissue renewal, and inflammation by suppressing phosphoinositide 3-kinase signaling. ABCD decreases the levels of inflammatory cytokines such as interleukins (IL-6 and IL-8) and increases the level of IL-1β, which is crucial for successful implantation [61]. These results provide more evidence that ABCD may have consistent efficacy across procedures involving different timings of infusion.

The reproductive outcomes of FET after intrauterine ABCD infusion were better in both groups. The CPR was 40% in the NEM group and 28.12% in the TEM group. The percentage of live births was 30% in the NEM group and 18.8% in the TEM group. Compared with those in the TEM group, the patients in the NEM group were more beta-HCG positive and had greater clinical pregnancy and live birth rates. However, this difference was not statistically significant. In total, 40.3% of the patients were beta HCG positive, and 24.2% of whom delivered live babies. Our IVF results are consistent with those of previous studies.

Maged et al. conducted a recent meta-analysis of both RCTs (886 individuals) and non-RCTs (732 participants) and reported that PRP treatment improved implantation rates, CPR, and LBR. A recent RCT demonstrated that PRP has a significant positive effect on endometrial vascularity, EMT, biochemical pregnancy rates (18.64%), and CPR (23.73%) [57, 62].

Fuji et al., among 99 FET cycles, reported findings similar to our findings in both the NEM and TEM groups. The rate of hCGR was 46.7%, the CPR was 41.0%, and the LBR was 36.2%. The quality of transferred embryos had no effect on the outcome of PRP-FET in terms of clinical pregnancy success when good-quality embryos of 4BB or higher with enlarged blastocysts on the fifth to sixth days after fertilization were used. This finding is in line with our findings, which revealed that the quality of the transferred embryos had no effect on ABCD-FET success or failure with respect to clinical pregnancy status. In another study including 50 patients with refractory TEM, the CPR was 50%, whereas the LBR was 42%. Additionally, the EMT significantly increased by an average of 1.6 mm after the administration of three doses of intrauterine ABCD infusion [58, 63].

Mehrafza et al. conducted a retrospective cohort study to examine the effects of ABCD and GCSF on pregnancy outcomes in RIF patients. The CPR was significantly greater in the ABCD group than in the GCSF group, at 40.3% and 21.4%, respectively [64]. A meta-analysis of ten studies (*n* = 1555; 775 cases, 780 controls) revealed that women treated with PRP had better pregnancy outcomes, including clinical and chemical pregnancy rates, implantation rates, LBR, and abortion rates, than controls did [65]. Other studies reported similar outcomes. [66–68].

The potential impact of ABCD infusion on reducing the occurrence of spontaneous abortion remains uncertain. Patients with TEM are much more likely to experience early pregnancy loss. A poorly prepared endometrium is believed to be unable to sustain a pregnancy [69]. According to Yuan’s study, the miscarriage rate was as high as 26.7% in patients who became pregnant with IVF and had an EMT of less than 8 mm [70]. In Liu’s trial, the rate of miscarriage increased by 1 mm for every 8 mm decrease in EMT [71]. The abortion rates were 20% in the NEM group and 9.4% in the TEM group. Both groups had the same number of abortions. This study shows that administering ABCD infusion directly into the uterus minimizes the risk of spontaneous abortion. Further research is needed to understand the mechanism by which ABCD reduces the likelihood of miscarriage. However, it may be claimed that the ABCD infusion aided the endometrium in attaining sufficient capability to maintain pregnancy.

However, despite the efficacy of ABCD infusion in reducing implantation failure rates, the ESHRE Working Group on RIF stated that there is insufficient evidence to fully justify its use; hence, further evaluation is needed [72]. Additionally, evidence suggests that ABCD is ineffective. ABCD is ineffective as an adjuvant treatment for patients who have RIF and a NEM (> 7 mm) and who are undergoing embryo transfer during IVF [73]. Another study revealed that ABCD infusion did not impact reproductive outcomes in people with a history of RIF and an EMT greater than 8 mm [74].

Aghajanzadeh et al. reported no significant differences in the CPR, chemical pregnancy rate, or ongoing pregnancy rate between the control and PRP-treated groups of 30 RIF patients. The limited sample size and the inclusion of women with thrombotic abnormalities in the study might have influenced the outcomes [75]. In another retrospective study, 15 patients with RIF and 39 with TEM (< 8 mm) showed no increase in EMT during the PRP cycle compared with the prior ET cycle [76]. A recent prospective study by Zargar et al. demonstrated that PRP had no effect on RIF patients. However, 80 women had a mix of fresh and frozen cycles (25% in the PRP arm had a fresh embryo transfer vs. 10% in the control group), casting doubt on the study’s findings. For better results, researchers could have employed fresh or FET cycles instead of a combination [77].

The negative outcomes obtained in these studies may be attributed to the small sample size and the lack of a clear description of the methods employed for preparing PRP, which significantly varied between these studies. To assess the potential benefits of PRP in the field of reproductive medicine, an extensive review of the protocols used in various studies is needed. The factors that affect the outcome of IVF in relation to PRP preparation include blood volume and platelet concentration, centrifugation force and duration, use of commercial kits, initial and final platelet concentrations, volume of PRP injected, timing of PRP injection in the patient’s cycle, timing of PRP injection after preparation, and frequency of injections. The primary distinction lies in the centrifugation stage, which is a critical element of the PRP preparation procedure. It is essential to optimize centrifugation processes to reduce variability in outcomes and achieve consistency. A crucial factor to consider when evaluating the given protocols is persistent failure to monitor platelet concentration at any stage of the treatment procedure [41, 78].

Furthermore, it is important to recognize that infertile patients who experience implantation failure are not homogeneous. Prior to ABCD treatment, performing a comprehensive assessment of immunological and inflammatory factors, as well as meticulously recording any signs of endometritis, is critical. To optimize the effectiveness of ABCD treatment, it is advisable to categorize patients on the basis of the likely reason for their implantation failure.

In the present study, we compared ABCD PRP with the conventional PRP utilized in prior studies. The method of extracting a concentrated form of growth factor from platelets provides several benefits compared with the use of conventional PRP. This method increases the concentration of growth factors, potentially improving their therapeutic benefits. Furthermore, the ability to selectively extract specific growth factors allows for more precise customization of therapy to cater to the individual requirements of each patient, thereby improving outcomes [40, 79]. By employing these growth factors in combination with an appropriate delivery system, it is feasible to accomplish controlled and prolonged release. The extraction procedure has the ability to mitigate the dangers of platelet activation, such as coagulation and inflammation, by removing lymphocytes [80]. Compared with PRP, isolated growth factors offer enhanced stability and convenience for storage, which frequently requires fresh preparation and immediate usage [81].

A comprehensive analysis conducted in 2020 revealed that for every one-unit increase in BMI, the likelihood of successful implantation after IVF was reduced by 2.2–4.3%. Pantasri et al. reported that obese women may have abnormal gene expression, which might lead to a less favorable window for implantation and increase the likelihood of RIF [82, 83]. Another factor to consider when defining RIF is the age of the mother. The likelihood of becoming pregnant decreases as a woman becomes older [84]. Older patients need to undergo more cycles of blastocyst transfer to obtain the same rate of successful implantation as younger women do [85].

The study by Fuji et al. aimed to investigate the parameters that influence the success of intrauterine autologous PRP infusion in patients with RIF. The explanatory variables chosen for the analysis were age, BMI, previous implantation failure, EMT, and embryo grading. Previous implantation failure was significantly associated with hCGR, CPR, and LBR in the RIF group but not in the TEM group. In the TEM group, age and BMI were weakly linked with the number of live births. Another study examined the parameters influencing the success of intrauterine PRP in the TEM group and reported that the number of previous uterine surgeries and thin EMT were significant predictors of pregnancy failure following PRP injection [58, 86]. However, our findings contrast those of Fuji et al. We found no significant associations between age, BMI, number of previous implantation failures, final EMT and embryo grading, and hCGR, CPR, or LBR in either group.

The results we obtained suggest that employing ABCD therapy improves IVF outcomes in patients with RIF. The outcome signifies a substantial enhancement in the ability of the endometrium to receive an embryo, rather than solely focusing on the process of endometrial growth. This treatment is specifically engineered to be exceptionally effective in treating infertility, where rapid regeneration is crucial for achieving the desired therapeutic outcomes. The primary benefit of ABCD growth factor concentrate is its ability to stimulate the secretion of growth factors at levels significantly higher than the body’s natural levels. This concentrate, obtained from the patient’s own platelet concentrates, has the ability to quickly initiate tissue regeneration and remodeling. This is particularly important in the field of infertility, where time is essential, and completing cycles successfully depends on fast tissue regeneration within a timeframe of less than 15 days [87, 88].

The ABCD growth factor concentrate offers several advantages, such as the capacity to derive multiple doses from a single blood collection and ensure consistent dosages. This approach provides ease for both reproductive care providers and patients, making it easier to incorporate into IVF protocols.

This study is limited by its small sample size, retrospective methodology, and single-center design. In addition, we defined a thin endometrium as a diameter of less than 8 mm, but some studies have suggested that a diameter of less than 7 mm is preferable for predicting reproductive success. Importantly, conclusions may vary depending on the definition. Furthermore, since many patients were referred to our center following unsuccessful IVF treatments conducted elsewhere, we lack detailed data on their previous implantation failures. Nonetheless, we believe that the results of this study are trustworthy since the investigation was carried out under a consistent methodology in a controlled setting to address the existence of other elements that might have influenced implantation failure.

## Conclusion

Despite the wide range of conventional treatment options available today, providing a practical and supported approach that can help clinicians improve RIF patient management is challenging. This retrospective cohort study was conducted to examine the effects of ABCD treatment on refractory NEM and TEM. The CPR and LBR achieved were 33.87% and 24.2%, respectively. The outcomes show that ABCD treatment improves beta HCG positivity, CPR, LBR, and EMT in both NEM and TEM patients. An improvement in the LBR could represent the most significant clinical result for RIF patients. In accordance with these findings, the intrauterine infusion of ABCD should be considered advantageous for patients who have had two failed FETs utilizing high-quality embryos. The current results may aid healthcare practitioners in determining whether ABCD infusion may be employed as a therapeutic intervention for refractory patients. Additional research is necessary to confirm the efficacy of ABCD and establish the most appropriate dosage and timing for its administration.

## Supplementary Information

Below is the link to the electronic supplementary material.

Supplementary file1 (DOCX 15 KB)

## Data Availability

De-identified patient data used for this study is available upon request.

## Code availability

No code was used for the analysis of the data.
