# Supplementary material for: Efficacy of intrauterine autologous blood cell derivatives in enhancing endometrial thickness and IVF outcomes for women with recurrent implantation failure: a retrospective cohort study
Source: J Assist Reprod Genet. 2024 Sep 5;41(10):2667–80. doi: 10.1007/s10815-024-03231-5 (PMC11535134; doi:10.1007/s10815-024-03231-5)
Supplement: Supplementary file 1 — Supplementary file1 (DOCX 15 KB) [file 10815_2024_3231_MOESM1_ESM.docx]

**Efficacy of Intrauterine Autologous Blood Cell Derivatives in Enhancing Endometrial Thickness and IVF Outcomes for Women with Recurrent Implantation Failure: A Retrospective Cohort Study**

**Supplementary file**

**Supplementary Table 1**: **Changes in endometrial thickness before and after an infusion of ABCD in the NEM and TEM groups**

| **EMT thickness** | **NEM group (n=30)**  **Median (IQR) Variance p value*** | **TEM group (n=33)**  **Median (IQR) Variance p value*** | **Total (n=63)**  **Median (IQR) Variance** |
| --- | --- | --- | --- |
| The day of first ABCD Infusion | 8.95 (1.22) 0.58 0.20 | 7.10 (1.30) 0.57 0.14 | 7.90 (1.9) 1.71 |
| The day of second ABCD Infusion | 9.70 (1.70) 1.78 0.12 | 8.00 (1.35) 1.56 0.08 | 8.60 (2.1) 2.54 |
| The day of third ABCD Infusion | 9.88 (2.00) 2.11 0.06 | 8.40 (1.30) 1.10 0.055 | 9.00 (1.8) 2.10 |

The median endometrial thickness and variance between the NEM and TEM groups were compared via Student's t test. *Normal distribution of the EMT across the NEM and TEM groups was calculated via the Kolmogorov–Smirnov test of normality.

Abbreviations: ABCD, autologous blood cell derivative; EMT, endometrial thickness; IQR, interquartile range; NEM, normal endometrium; SD, standard deviation; TEM, thin endometrium
